# Cardiopulmonary exercise testing after cardiac resynchronization therapy: unit harmonization and effect-size methods require clearer reporting

**DOI:** 10.1093/ehjopen/oeag037

**Published:** 2026-02-21

**Authors:** Shoaib Majeed, Jie Tu

**Affiliations:** Department of Clinical Medicine, Qiqihar Medical University, Qiqihar 161006, Heilongjiang, China; Department of Neurology, Qiqihar Medical University, Qiqihar 161006, Heilongjiang, China; Department of Clinical Medicine, Qiqihar Medical University, Qiqihar 161006, Heilongjiang, China; Department of Neurology, Qiqihar Medical University, Qiqihar 161006, Heilongjiang, China

**This correspondence refers to ‘The effect of cardiac resynchronization therapy on functional capacity based on cardiopulmonary exercise testing: a systematic review and meta-analysis’, by J. Solano *et al*. https://doi.org/10.1093/ehjopen/oeaf176.**

**The authors’ response is available at https://doi.org/10.1093/ehjopen/oeag035.**

We read with great interest the systematic review and meta-analysis by Solano and colleagues assessing the effect of cardiac resynchronization therapy (CRT) on functional capacity using cardiopulmonary exercise testing (CPET).^1^ The authors included 14 studies (*n* = 858) and reported pooled improvements in peak VO_2_, anaerobic threshold, and ventilatory efficiency, alongside substantial heterogeneity, and evidence suggestive of publication bias for peak VO_2_.^1^

Several reporting and analytical issues may materially influence interpretation of the pooled estimates.

First, peak VO_2_ unit harmonization appears inconsistent. Table 1 labels mean baseline pVO_2_ as mL/kg/min, yet at least two entries are marked with an asterisk and appear to be reported in mL/min (absolute VO_2_).^1^ As absolute and weight-normalized VO_2_ are not interchangeable without explicit conversion,^2^ mixing metrics may contribute to heterogeneity and *reduce* clinical interpretability. Separate analyses for absolute vs. weight-normalized VO_2_ (or clear conversion where feasible), together with a sensitivity analysis excluding non-mL/kg/min studies, would strengthen confidence in the pooled peak VO_2_ estimate.^1^

Second, the methods describe meta-analyses comparing baseline vs. follow-up means within the same cohorts.^1^ For pre–post (paired) designs, the sampling variance depends on the within-participant correlation between baseline and follow-up measurements; if this correlation is not incorporated, study weights and confidence intervals may be misestimated.^3,4^ Clear reporting of the exact pre–post effect-size method used, any assumed correlation, and sensitivity analyses across plausible correlation values would improve transparency.^3,4^

Third, the review includes two randomized controlled trials (RCTs) in addition to cohort studies.^1^ Where available, controlled RCT contrasts (between-group difference in change) are generally preferable to within-arm pre–post changes alone, as they better isolate intervention effects from time trends and regression to the mean.^4^ Presenting controlled RCT estimates and an RCT-only synthesis (where data permit) would improve causal interpretability.^4^

Finally, heterogeneity is very high for key outcomes (for example, *I*^2^ reported as 94% for peak VO_2_ and 96% for anaerobic threshold).^1^ With such variability, additional robustness checks, including leave-one-out analyses, subgrouping by CPET modality and follow-up timing, and prediction intervals, would help readers understand how effects may vary across settings.^1^ The reported funnel plot asymmetry and significant Egger’s test for peak VO_2_ (*P* = 0.014) further support cautious interpretation of pooled effects.^1^

This review addresses an important clinical question and provides a helpful synthesis of CPET changes after CRT.^1^ Clarifying unit handling and effect-size methods, and adding targeted sensitivity analyses, would strengthen the reliability and clinical interpretability of the conclusions.

## Data Availability

No new data were generated or analysed in this study.
